# Contemporary Management of Chronic Prostatitis

**DOI:** 10.7759/cureus.20243

**Published:** 2021-12-07

**Authors:** Ahmed S Khattak, Nicholas Raison, Arie Hawazie, Azhar Khan, Oliver Brunckhorst, Kamran Ahmed

**Affiliations:** 1 Urology, King's College Hospital, London, GBR; 2 MRC Centre for Transplantation, Guy's Hospital Campus, King's College London, King's Health Partners, London, GBR; 3 Urology, Queen's Hospital, London, GBR; 4 Urology, King's College London, London, GBR; 5 Urology, King's College Hospital NHS Foundation Trust, London, GBR

**Keywords:** chronic prostatitis, pelvic pain, management, prostate, cpps

## Abstract

Chronic prostatitis (CP) is a common condition, yet remains a challenge to treat in clinical practice due to the heterogeneity of symptoms.

The aim of this article is to undertake a narrative review using key research papers in this field in order to develop a treatment algorithm and research recommendations for the management of type II and type III prostatitis taking a broader look at interventions beyond those recommended in the European Association of Urology Guidelines.

A search was performed using multiple databases and trial registries with no language restrictions. Searches were completed on March 1, 2021, with a focus on randomized controlled trials (RCTs), meta-analyses, and systematic reviews. However, in areas with a dearth of such studies, we included case series and observational studies, thus allowing us to assess current levels of evidence and areas of potential research.

We identified and reviewed 63 studies. The level of evidence and the quality of trials were assessed and reported. Research recommendations, where applicable, were also highlighted.

CP/chronic pelvic pain syndrome (CPPS) is a heterogenous term referring to diverse symptomology that requires tailored treatments depending on the patients’ complaints. After a review of the evidence available, we present a treatment algorithm that is based on the much-discussed UPOINT (urinary symptoms, psychosocial dysfunction, organ-specific findings, infection, neurologic/systemic, and tenderness of muscles) framework. Future studies should focus on multimodal therapy based on such frameworks and provide the future direction of this complex condition.

## Introduction and background

Prostatitis is a common condition with an incidence of 4.5%-9% [1]. However, despite its prevalence, the syndrome remains a challenge in clinical practice. In part, this relates to the heterogeneous definition of prostatitis. The National Institutes of Health (NIH) classification system set out four syndromes that come under the umbrella of prostatitis.

Chronic prostatitis/chronic pelvic pain syndrome (CP/CPPS) is defined by Category II and Category III of the NIH classification system and is particularly difficult to treat, with a recurrence rate of up to 50% [2]. Table 1 illustrates the NIH classification system of prostatitis as published in the journal of the American Medical Association, which has been in use since 1999 [3].

**Table 1:** NIH classification of prostatitis Source: [3] NIH=National Institutes of Health; EPS=expressed prostatic specimen - fluid collected during a prostatic massage; VB3=voided bladder specimen 3 - first 10 ml of urine collected after EPS

Category	Description
I	Acute bacterial prostatitis is an acute infection of the prostate
II	Chronic bacterial prostatitis is a recurrent infection of the prostate
III	Chronic nonbacterial prostatitis/chronic pelvic pain syndrome (CP/CPPS) when there is no demonstrable infection. Subgroups of this class are: A. Inflammatory CP/CPPS when leukocytes are found in the semen, expressed prostatic secretions (EPS) or urine obtained after prostate massage (voided bladder urine 3 (VB3)); B. Noninflammatory CP/CPPS when no evidence of inflammation is found in the semen, EPS, or VB3
IV	Asymptomatic inflammatory prostatitis when there are no subjective symptoms, but white cells are found in prostate secretions or in prostate tissue during an evaluation for other disorders

The pathogenesis of CP/CPPS remains poorly understood. Infection and anatomical abnormalities have been implicated in this syndrome. High-pressure voiding dysfunction, intraprostatic ductal reflux, and autoimmune and neuromuscular mechanisms are all thought to play a role in the etiopathogenesis of CP/CPPS. Ultimately, the cause of CP/CPPS is often multifactorial, which leads to such diverse symptomology and presentation.

CP/CPPS is defined by pelvic pain lasting at least three of the prior six months and is often coupled with lower urinary tract symptoms (LUTS) [3]. Pain is usually felt in the pelvis, the lower abdomen, the back, and the genitals. CP/CPPS is commonly associated with other syndromes such as fibromyalgia, irritable bowel syndrome (IBS), depression, and stress [4].

The severity of CP/CPPS symptoms should be assessed using the validated NIH Chronic Prostatitis Symptom Index (CPSI), a nine-question survey covering three areas: pain, urinary symptoms, and quality of life (QoL) [5].

CP/CPPS requires a holistic treatment approach. A full physical examination with a focus on a digital rectal exam (DRE) must be performed to characterize the prostate and the origin of pain. Occasionally, the prostate itself is not the offending organ in CP/CPPS but it is rather pelvic floor tenderness/spasm, which will require a different treatment approach [6]. A prostate-specific antigen (PSA) should be performed if the patient is at risk of prostate cancer or if an examination has suggested it.

Urodynamic studies should only be considered in the presence of LUTS as can cystoscopy if clinically indicated.

**Figure 1:**
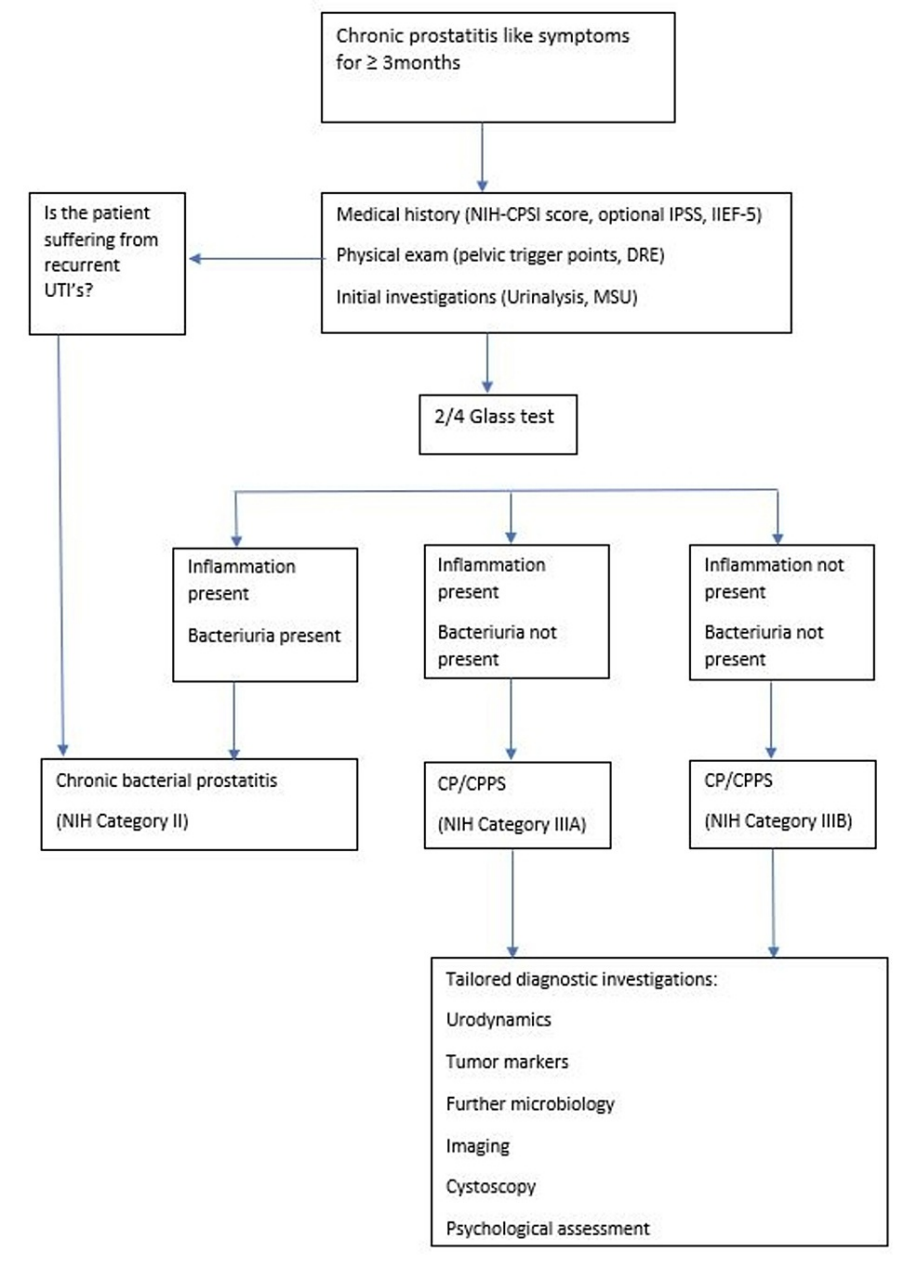
Diagnostic algorithm Adapted from Magistro et al. [7] IIEF=International Index of Erection Function; IPSS=International Prostate Symptom Score; DRE=digital rectal examination; MSU=midstream specimen of urine; UTI=urinary tract infection

Figure 1 illustrates a diagnostic algorithm as suggested by Magistro et al. for CP/CPPS [7].

The gold standard diagnostic approach for chronic bacterial prostatitis is a urine culture with the Meares-Stamey four-glass approach (Table 2). The samples are sent for culture to identify pathogens and leukocytes. Given that this diagnostic test is relatively onerous to conduct, a two-glass approach may be used after a prostatic massage, with equivalent efficacy [8].

**Table 2:** Meares-Stamey four and two-glass test protocol Adapted from Rees et al. [8] Summary: To find a bacterial cause to symptoms in CP/CPPS, the four-glass (Meares-Stamey) test is considered the "gold standard" for diagnosis, whereby voided bladder (VB) urine (VB1, VB2, and VB3) and EPS samples are taken for culture and microscopic analysis. The two-glass test (VB2 and VB3) has been shown to offer similar diagnostic sensitivity to the four-glass test while other studies advocate urethral swab plus post-prostatic massage urine analysis. The test used often is left to local protocols or clinician preference. VB1=voided bladder specimen 1 - first 10 ml of urine; VB2=voided bladder specimen 2 - second 10 ml of urine taken after the passage of 100 ml of urine; EPS=expressed prostatic specimen - fluid collected during a prostatic massage; VB3=voided bladder specimen 3 - first 10 ml of urine collected after EPS, CP/CPPS=chronic prostatitis/chronic pelvic pain syndrome

Steps of test	Included in the four-glass test	Included in the two-glass test
VB1 (represents the urethra)	x	
VB2 (represents the bladder)	x	x
EPS (represents the prostrate)	x	x
VB3 (represents the prostatic urethra)	x	

Aims

We aimed to undertake a narrative review using the key research papers in this field in order to develop a treatment algorithm for managing type II and type III prostatitis. This review encompassed a broader look at interventions beyond those recommended in the European Association of Urology guidelines.

Methods

The study group performed a search using multiple databases and trial registries with no language restrictions. Searches were completed on March 1, 2021, with a focus on randomized controlled trials (RCTs), meta-analyses, and systematic reviews. However, in areas with a dearth of high-quality evidence, we included case series and observational studies, thus allowing us to assess current levels of evidence and areas of potential research. We excluded studies with incomplete data or those not dealing exclusively with type II or III prostatitis. Where data was incomplete, not recorded, or unclear, we analysed available data in the study, analysed the study based on that and assessed its viability for inclusion. Where possible, trials and studies were analyzed with risk-of-bias tools. The search terms used were as follows: Chronic Prostatitis, Prostatitis, CPPS/CP, Management, Treatment, Surgery, Prostatectomy, TURP, Pharmacology, Medication, Alpha-Blockers, Antibiotics, NSAIDs, Anti-Inflammatory, BOTOX/Botulinum-Toxin, Antibiotics, Anti-Depressants, 5-ARI, Finasteride, Phytotherapy, Supplements, ESWL/Extra-Corporeal Shock Wave Lithotripsy, Traditional Chinese Medicine, Acupuncture, Electro-Acupuncture, Prostatic Massage, Physical Activity, Physiotherapy, Lifestyle.

**Figure 2:**
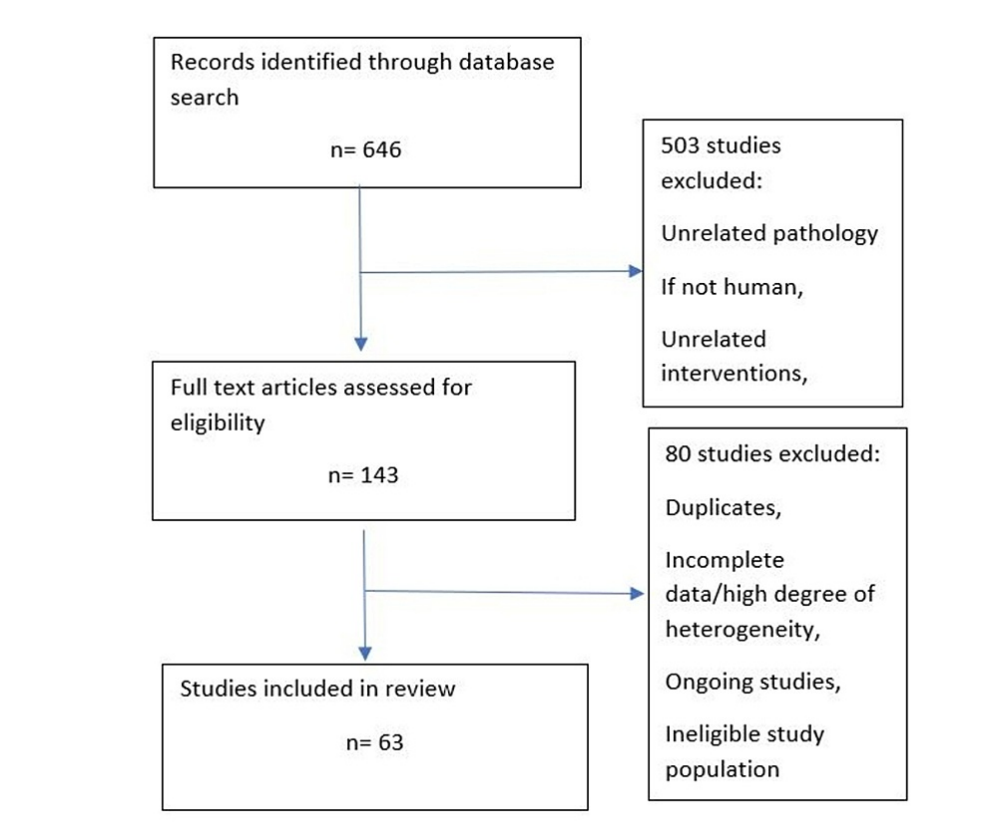
Flow chart for selection of studies

One author independently reviewed articles and studies, interpreted the data and assessed the quality of evidence. The primary outcome was prostatitis symptoms as measured by the NIH-CPSI assessment scores and adverse events. Secondary outcomes were QoL, sexual dysfunction, urinary symptoms, and adverse outcomes. Figure 2 displays the flow chart for the selection of studies and reasons for exclusion.

## Review

Non-pharmacological treatments

There have been numerous non-pharmacological and non-surgical treatments trialled to treat CP/CPPS. For the purpose of this study, we reviewed RCTs and systematic reviews found via EMBASE, Medline, MESH, and Cochrane database searches; where none were found, we reviewed the highest level of evidence available. The available evidence was from 1994 to 2020.

Acupuncture

Acupuncture is one of the most common non-pharmacological therapies and is used for a variety of chronic conditions [9]. It is thought to work via sensory nerve stimulation, augmenting pain perception [10-11]. Traditional Chinese medicine (TCM) philosophy hypothesizes that the augmentation of pain perception is achieved through the regulation and redistribution of neurovascular flow (Qi) in the body, which thereby improves any neuromuscular dysfunction [12]. Western medical theory suggests that local needling in acupuncture treatment may stimulate tissue healing and give rise to local pain relief through the modulation of pain reflex arcs and the release of neuropeptides and local endorphins [13-14].

Different styles of acupuncture have been developed, including TCM, non-insertion acupuncture (the use of blunt needles on trigger points around the body), and auricular, scalp, and hand (needling different parts of each location that represent different organs). Although TCM is the most commonly implemented type of acupuncture, all types of acupuncture were included in this study.

A global overview of the studies in Table 3 lends credence to the efficacious use of acupuncture in cases of CP/CPPS which illustrates the mean difference in NIH-CPSI scores. There is evidence of improvement in NIH-CPSI scores at short-term follow-up, particularly in pain domains (Table 3). However, further benefits of acupuncture versus a sham procedure were not found with any sort of statistical significance [15-18].

**Table 3:** Results of acupuncture vs sham procedure *24 patients were compared against each other (sham procedure vs acupuncture) f/u = follow-up

Study	Number of Subjects	Acupuncture Difference	Sham Difference	P-Value
Lee et al 2008 [16]	90	-10.3 +/-7.67 (10 weeks f/u)	-5.3+/-7.67 (10 weeks f/u)	0.03
Lee et al 2009 [17]	24*	-9.5+/-3.7 (6 weeks f/u)	-3.5+/-3.6 (6 weeks f/u)	<0.001
Sahin et al 2015 [18]	100	-15.94+/-3.03 (8 weeks f/u)	-9.42+/-5.81 (8 weeks f/u)	<0.001
Qin et al 2018 [15]	68	-10.8+/-2.5 (8 weeks f/u)	-5.1+/-1.5 (8 weeks f/u)	<0.001

Lee et al. illustrated a 73% positive response rate in NIH-CPSI scores compared to 47% in the sham group [16]. Longer-term studies will be required to validate whether these improvements are maintained but, as Franco et al. commented in their Cochrane review, acupuncture likely leads to a reduction in prostatitis symptoms compared with a sham procedure [19]. No study mentioned any adverse events. Table 4 illustrates the mean difference when specifically looking at pain, highlighting its potential in this specific domain:

**Table 4:** Acupuncture vs sham procedure NIH-CPSI pain scores f/u=follow-up, NIH-CPSI=National Institutes of Health-Chronic Prostatitis Symptom Index

Study	Mean Pain Score Pre-Acupuncture (Sham score)	Mean Pain Score Post-Acupuncture (Sham score)	P-Value
Lee et al 2008 [16]	11.4+/-3.4	7.0+/-4.5 (8.0+/-4.8) 6 weeks f/u	0.12
Lee et al 2009 [17]	12.2+/-2.5 (11.8+/-2.7)	7+/-1.9 (10.2+/-1.8) 6 weeks f/u	<0.001
Sahin et al 2015 [18]	13.2+/-2.3 (13.0+/-2)	7.16+/-1.81 (10.41+/-3.71) 24 weeks f/u	0.001
Qin et al 2018 [15]	12.4+/-2.8 (11.4+/-2.1)	5.9+/-1.8 (8.4+/-1.8) 32 weeks f/u	<0.001

When compared with medications, Lee et al. (in the third arm of their study) and Kucuk et al. found that acupuncture may also lead to a clinically meaningful reduction in prostatitis symptoms compared with standard medical therapy (mean difference -6.05, 95% CI (confidence interval) -7.87 to -4.24, two studies, 78 participants) with no real difference seen between the use of electroacupuncture and standard acupuncture [16,20].

The quality of evidence of all the studies analyzed is low and therefore further studies will be required to assess true efficacy versus medication. Masking was a clear issue, especially when compared to medication. Beyond that, notwithstanding a degree of heterogeneity (e.g., electroacupuncture as an intervention or traditional needlepoint acupuncture and other variants coupled with moxibustion etc.), acupuncture holds value in the treatment of CP/CPPS.

Lifestyle Modification

By its very nature, lifestyle modification involves a broad, holistic, non-pharmacological approach requiring the initial identification of pertinent modifiable risk factors and subsequent stringent adherence to assessing change. Thus, few studies have been performed in this domain.

One such study was performed by Gallo in which 100 patients with CP/CPPS were recruited [21]. A literature search was performed to identify risk factors associated with CP/CPPS, as well as broader risk factors associated with interstitial cystitis/bladder pain syndrome (which were deemed related conditions by the study team). A lifestyle guideline was created, and patients were randomized to adhere to the guideline and a prescribed nonsteroidal anti-inflammatory drug (NSAID) or simply to an NSAID.

The results of the study illustrated a statistically significant (p<0.0001) reduction in the NIH-CPSI score, from 22.1 to 8.1, in the intervention group versus 21.9 to 17.6 in the control group. However, the quality of the data was low. There was no concealment, leading to a high risk of detection and selection. Additionally, as the study was not blinded, performance bias could well have been an issue. The study relied on patients simply answering a binary question concerning adherence to lifestyle changes, lending itself to reporting bias. The incidence of adverse events was also not reported, and there was a significant dropout from the intervention group (22%), suggesting difficulty in patient adherence [21].

It is the view of the study group that lifestyle changes should be suggested given the potentially modifiable risk factors associated with CP/CPPS and that further research in this area may help direct non-pharmacological treatments.

Physical Activity

Giubilei et al. conducted a study that originally included 103 participants with CP/CPPS aged 20 to 50 and compared physical activity versus a control intervention in patients who had lost confidence in pharmacological treatments [22]. At the end of the 18-week follow-up period, 76 participants remained for analysis. There was a suggestion of benefit in the intervention group, with a total reduction in the NIH-CPSI (Italian Version) score from 21.9 +/- 6.5 to 14.6 +/-4.9. The net change was -7.4 +/- 6.2, with a p-value of 0.0001, but with such a large confidence interval, definitive conclusions cannot be made until larger-scale, longer-term studies are performed.

Although both arms of the study showed improvements in anxiety scores, there was no difference in net change between them (6.2, with a wide confidence interval of +/-6.9), and despite improvements being recorded in all measured outcomes (pain, urinary symptoms, QoL (quality of life), visual analogue scale (VAS)-pain score, anxiety and depression), the wide confidence interval yielded indeterminate conclusions regarding urinary symptoms, QoL, anxiety, and depression [22].

No recording of adverse events was made, but with the large dropout rate in the intervention arm, it can be assumed that the intervention was not well-tolerated the longer the trial lasted. Beyond that, the study was at high risk of performance and detection bias, as true blinding was not achievable.

All in all, this study did not suggest physical activity as an evidence-based treatment for CP/CPPS. That said, however, larger, longer-term studies would be helpful, especially in the context of the overall health benefits of physical activity.

Prostatic Massage

Prostatic massage had been theorized to ameliorate chronic prostatitis even before effective antimicrobial agents became available for CP/CPPS. Indeed, prostatic massage was once considered a mainstay of treatment. The basis of this therapy centres around presumed prostatic congestion, which would be relieved by expelling inflammatory cells and fluid from the obstructed acini of the gland [23]. The true mechanism, however, remains subject to conjecture, with physical disruption of a biofilm or relief of pelvic muscle spasm also suggested, as well as improved blood flow to the gland [24].

Three studies were identified that dealt with prostatic massages. However, one Chinese-language paper had to be excluded due to a lack of data and the inaccessibility of the authors. Nonetheless, the paper suggested that those who received a prostatic massage in combination with TCM versus traditional oral Chinese medicine only had lower sub-scores for pain (-3.3 +/-1.12), urinary symptoms (-2 +/-0.79), and QoL (-.2.9 +/-0.68). Further conclusions and the quality of evidence are, however, difficult to assess [25].

Ateya et al. compared prostatic massage in combination with antibiotics in 25 patients versus only antibiotics in 19 patients with NIH type IIIb chronic nonbacterial prostatitis (they also had another arm of the study that compared NIH type II patients) [25]. The authors concluded that despite some improvements in total NIH-CPSI scores in both groups, a greater difference was seen with the use of antibiotics alone, with a total score decrease of 12.4 +/-7.1 versus 11.3 +/-8.2. Hence, prostatic massage could not be deemed an effective treatment for CP/CPPS [26].

Beyond these two RCT trials, two other studies designed as case series have been performed, the largest of which - Shoskes et al. - used prostatic massage with antibiotics as an intervention. The study found that patients with NIH category IIIb CP/CPPS had no improvement at all, and only 24% of NIH class IIIa patients had complete improvement (defined as greater than 50% improvement in the NIH-CPSI score) by the end of the two-month treatment period [27]. Nickel et al. published a similar case series in which 26 highly motivated patients travelled to the Philippines for treatment at their own expense and exhibited mild improvement [28]. Table 5 highlights the structure and results of each of the studies:

**Table 5:** Results summary of assessed studies regarding prostatic massages RCT=randomized control trial; TCM=traditional Chinese medicine

Study	Type of Study	Number of Subjects	Intervention	Results	Adverse Events
Ateya et al 2006 [26]	RCT	44 patients with NIH-category IIIb prostatitis	Prostatic Massage (three times per week) with antibiotics vs antibiotics only	No significant difference between groups	Not recorded
Shen et al 2006 [25]	RCT	72 patients with NIH-category IIIb prostatitis	TCM vs TCM + prostatic massage	Statistically significant (p <0.005) falls in pain, micturition and QoL scores	One epididymitis, seven Mycoplasma culture positive
Nickel et al 1999 [23]	Case series	26 Patients with CP/CPPS	Prostatic massage (three times per week) with antibiotics vs antibiotics only	Statistically significant improvement in QoL but nil in pain, urinary or sexual symptoms	Not recorded
Shoskes et al 1999 [27]	Case series	19 patients with NIH-category IIIa/b prostatitis	Prostatic massage (two times per week) with antibiotics vs antibiotics only	No significant Improvement	Not recorded

The overall level of evidence was low, with a high risk of detection and performance bias. No clear improvement was evident from the studies, and there has been little recent research or comment regarding prostatic massage (either as an individual treatment or in combination) in the treatment of CP/CPPS. The authors of this review cannot, therefore, recommend prostatic massage as a treatment for CP/CPPS.

Extracorporeal Shockwave Therapy (ESWL)

Primarily used in the treatment of urolithiasis, ESWL has been successfully implemented in chronic conditions such as epicondylitis, plantar fasciitis, and even diabetic wounds [29]. Its analgesic effect in the treatment of urolithiasis was discovered by chance during stone treatment and has since been studied in the treatment of various urological conditions, from Peyronie’s disease to erectile dysfunction to CP/CPPS [30].

We found five RCTs (shown in Table 6) that compared ESWL with sham procedures [29-33]. In the short-term follow-up, there were marked reductions in total NIH-CPSI scores.

**Table 6:** Results of assessed studies - total NIH-CPSI scores mean difference in scores of low-intensity ESWL vs control group at four weeks Adapted from Yuan et al [34] ESWL=extracorporeal shockwave lithotripsy; NIH-CPSI=National Institutes of Health-Chronic Prostatitis Symptom Index Negative scores favour ESWL.

Study	Low Intensity-ESWL Mean NIH-CPSI Score (Standard Deviation)	Total Subjects	Control Group Mean NIH-CPSI Score (Standard Deviation)	Total Subjects	Mean Difference (95% Confidence Interval)
Zimmerman et al 2008 [29]	19.93 (3.18)	30	24.77 (3.07)	30	-4.84 (-6.42 to -3.26)
Pajovic et al 2016 [30]	10.16 (3.99)	30	16.8 (9.03)	30	-6.64 (-10.17 to -3.11)
Salama et al 2018 [31]	15.8 (3.54)	20	21.3 (3.15)	20	-5.70 (-7.78 to -3.62)
Vahdatpour et al 2013 [32]	21.4 (2.7)	20	24 (2.8)	20	-2.60 (-4.30 to -0.90)
Zeng et al 2012 [33]	21.9 (3.3)	40	29.8 (3.6)	40	-7.90 (-9.41 to -6.39)

After 12 weeks of follow-up, Salama et al. found a statistically significant decrease in total NIH-CPSI scores of -16.05 (CI -18.11 to -12.99) [31], and significant decreases were also observed in all other studies up until 12 weeks as seen in Table 7:

**Table 7:** Results of assessed studies- total NIH-CPSI Scores mean difference in scores of low-intensity ESWL vs control group at 12 weeks Adapted from Yuan et al. [34] Negative scores favour ESWL. ESWL=extracorporeal shockwave lithotripsy; NIH-CPSI=National Institutes of Health-Chronic Prostatitis Symptom Index

Study	Total Subjects	Mean Difference between Groups (95% Confidence Interval)
Zimmerman et al 2008 [29]	60	-5.30 (-6.94 to -3.66)
Pajovic et al 2016 [30]	60	-4.47 (-7.60 to -1.34)
Salama et al 2018 [31]	40	-16.05 (-18.88 to -13.22)
Vahdatpour et al 2013 [32]	40	-7.50 (-8.95 to -6.05)
Zeng et al 2012 [33]	80	-11.30 (-12.70 to -9.90)

Table 8 shows the weighted scores in three assessed domains (QoL, pain, urination) as per the NIH-CPSI scores.

**Table 8:** Weighted sub-scores at 12 weeks Source: [34] QoL=quality of life

Study	QoL Score	Pain Score	Urinary Score
Pajovich et al 2016 [30]	-2.03 +/- 1.59	-4.27 +/-1.88	-0.73 +/- 0.62
Salama et al 2018 [31]	-4.95 +/- 1.33	-8.20 +/-1.36	-2.90 +/-1
Vahdatpour et al 2013 [32]	-1.70 +/- 0.53	-4.20 +/- 0.80	-1.70 +/- 0.87
Zeng et al 2012 [33]	-4.20 +/-0.77	-5.70 +/-0.68	-0.40 +/- 0.44
P-Value	<0.00001	<0.00001	<0.0001

The longest follow-ups at 24 weeks post-treatment by Pajovic et al. still showed a statistically significant difference of -3.53 (CI -5.07 to -1.99). However, Vadhatpour et al. did not highlight a statistical difference in scores: -0.016 (CI -0.95 to 0.63) [30,32].

These differences were seen across the sub-scores of QoL, pain, and urinary symptoms and appeared to be well tolerated, with Zimmerman et al. reporting improvements in erectile dysfunction by the International Index of Erection Function (IIEF) scale (mean difference 3.34, 95% CI 2.68 to 4.00) [29].

ESWL has shown good efficacy for the treatment of CP/CPPS up to 12 weeks of follow-up. At 24 weeks, the difference seems to become less clear and may suggest that a second course of therapy may help. ESWL should be considered as part of the treatment arsenal when treating CP/CPPS.

Transrectal Thermotherapy (TRT)

TRT was originally developed for the treatment of benign prostatic hyperplasia (BPH). However, its application has broadened to include CP/CPPS. The mechanism of action of TRT is not well-elucidated but it is thought to be related to the reduction in oxygen-free radicals. Initial trials dealing with the transurethral delivery of heat yielded good results and had the benefit of allowing for higher target temperatures. However, adverse events, such as dysuria, hematuria, impotence, UTI, and pain, led to the evaluation of TRT [35].

Three different regimens (once weekly for four weeks, once weekly for six weeks, and two sessions weekly for three weeks) were studied by Montorsi et al. in 54 patients [36]. All of the recruited patients completed the study with no worsening of symptoms noted in any patient and a statistically significant improvement reported in voided volume and peak flow rate and marked improvement observed in QoL scores in all groups (although this was not statistically significant) [36].

When compared to medical therapy, Gao et al. showed that TRT seemed to improve prostatitis symptoms with a mean difference of -2.63 (95% CI -4.07 to -1.19) while Yoo et al. illustrated a mean difference of -1.80 (95% CI -5.12 to 1.52). However, when the weighted cumulative analysis was performed, the resulting p-value of 0.65 calls into question the validity of any conclusion that can be drawn from these studies [35,37].

When TRT was analyzed as add-on therapy, the mean difference rose in both studies: -4.59 (95% CI -5.99 to -3.19) in Gao et al. and -2.73 (95% CI -6.33 to 0.87) in Yoo et al. The p-value was lower (0.35) but was still not statistically significant [35,37].

The overall quality of evidence was low, with obvious issues regarding allocation, concealment, and performance bias. No serious adverse events were noted with transient hematuria, as noted in Montorsi et al. [35-36]. Montorsi et al. also noted two urinary tract infections (UTIs) and one episode of hemospermia [36].

The TRT intervention may be helpful in cases of CP/CPPS but further research is required to elucidate its true benefit and to quantify adverse events.

Physiotherapy

There is a dearth of quality evidence regarding physiotherapy and CP/CPPS. This is surprising given the potential availability of physiotherapists in surgical departments and the amount of literature that has highlighted the potential crossover in terms of the pelvic floor and prostatic pathology [38-39]. One case series of two patients by Van Alstyne et al. was too low in quality to be subject to statistical analysis; however, anecdotally, it highlighted the potential benefits that can be achieved with physiotherapy. A drop from 25 to 0 on the NIH-CPSI score in one patient and from 29 to 21 in another was achieved with supervised pelvic floor, flexibility, and aerobic exercises [40].

This is certainly an area for potential research and larger-scale trials in the future but cannot be recommended on the basis of currently available evidence. 

Surgical/invasive treatments

Four papers were found pertaining to surgery and CP/CPPS treatment: three papers dealing with prostatectomy as a treatment and one focusing on transurethral resection of the prostate (TURP) [41-44].

Radical Prostatectomy

Focusing on prostatectomies, four papers were identified. All of these papers (listed in Table 9) were cases series, thus representing a low level of evidence. One of these studies had to be excluded as the patient was later diagnosed with cancer on histology.

**Table 9:** Overview of results of radical prostatectomies in patients with NIH-CPSI III CP= chronic prostatitis, NIH-CPSI=National Institutes of Health-Chronic Prostatitis Symptom Index

Study	Number of Subjects	Characteristics	Results	p-value
Frazier et al 1992 [41]	5	CP (no NIH categorization)	Three patients had complete resolution of symptoms, two had mild residual discomfort	Not recorded
Krongrad et al 2010 [42]	6	NIH category III	All six patients had complete resolution of symptoms	<0.05
Chopra et al 2014 [43]	4	NIH category III	The median decrease in score was 23.5 (range 13-33)	Not recorded

The results all highlight improvements in symptoms among patients; however, there was a very high risk of performance bias, lack of concealment, and detection bias.

When reporting their results, Krongrad et al. noted improvement in inflammatory bowel disease (IBD) and generalized musculoskeletal (MSK) pain. Erectile function was affected more obviously, with poorer recovery in older patients [42]. No other complications were found in participants. It should be noted that all patients had undergone previous medical/alternative treatments with little improvement. Four participants had undergone TURP.

Postoperative erectile dysfunction affected three of the four patients in Chopra et al., which would be unacceptable for some patients [43].

Therefore, in selected patients, prostatectomy may provide a definitive treatment; however, this must be balanced against the risks of this procedure, which may be unacceptable for many patients, coupled with the fact that complete resolution may not be achieved, as illustrated by Frazier et al. [41]. As such, this treatment remains the last resort in the majority of cases.

Transurethral Resection of the Prostate (TURP)

TURP has historically been used for the relief of LUTS, with limited application in prostatitis. One case report was identified that focused on CP/CPPS, but it clearly suffered from concealment, performance, and detection bias. The overall quality of evidence was low, with very limited scope for recruitment, but it did highlight a potential treatment option. Kagawa et al. underscored the expected improvements in flow rates achieved in their case study by implementing TURP (max flow rate 12.4 ml/s to 29.8 ml/s), which should be considered when tailoring patient care [44]. However, as Krongrad et al. suggested in their case series, TURP may not be an effective treatment in CP/CPPS, as four of the six selected patients had undergone TURP prior to prostatectomy. Thus, based on the available evidence, TURP cannot be recommended as part of definitive treatment for CP/CPPS.

Overall, surgery may offer a previously unachievable level of relief in specific patients, especially in the context of modern laparoscopic techniques, but the choice of patient is key. Ultimately, this treatment should not form part of the mainstream management of CP/CPPS.

Medical treatments

Alpha-Blockers

Alpha-blockers inhibit type 1 alpha-adrenergic receptors and smooth muscle contraction located in the bladder, neck, prostate, and ureters. Via this mechanism of action, alpha-blockers provide treatment options for various conditions, from symptomatic BPH to hypertension to ureteric stones [45]. The basis upon which alpha-blockers are used in CP/CPPS centres around their efficacy in LUTS [46]. An anti-inflammatory effect has been suggested by some to be part of the mechanism by which alpha-blockers exert a therapeutic effect, with rat models showing reduced neurogenic inflammation when exposed to alfuzosin [45].

The 20 studies analyzed in this regard compared alpha-blockers to either a placebo or no additional treatment and were the same studies identified by Franco et al. in their review of alpha-blockers [47].

From the 18 studies that followed patients for up to six months, a mean difference in NIH-CPSI scores of -5.01 with a 95% CI of -7.41 to -2.61 was found [48-65]. This suggests a potential improvement in prostatitis scores, which was represented across all sub-scores (pain, urinary symptoms, and QoL).

Of the agents trialled, tamsulosin effected the biggest decrease in NIH-CPSI scores at short-term follow-up (302 patients, mean difference -5.89, 95% CI -13.16 to 1.38, p-value 0.11). Tamsulosin also had the second biggest effect on pain (260 patients, mean difference -3.17, 95% CI -6.31 to -0.31, p-value 0.05) [51], [57-59], but terazosin had a larger observed effect on pain (289 patients, mean difference -3.86, 95% CI -6 to -1.73, p-value 0) [48-52].

The biggest improvement in urinary function was seen with the use of terazosin (289 patients, mean difference -2.07, 95% CI -3.79 to -0.34, p-value 0.02), with a total improvement of urinary function across all six agents and 1,243 assessed participants of -1.48 (95% CI -2.29 to -0.66, p-value 0) [47-51].

In the longer term, the mean difference with alpha-blockers was observed as -5.6 (95% CI -10.89 to -0.32), with doxazosin, and terazosin being the most effective (mean difference -9.7, 95% CI -10.89 to -9.15, p-value<0.0001 and -7.76, 95% CI -10.9 to -4.62, p-value <0.0001, respectively).

The main adverse events (postural hypotension, dizziness, retrograde ejaculation, heartburn) had a risk ratio of 1.60 with a 95% CI of 1.09 to 2.34 and a p-value of 0.02. The agents with the highest risk ratio were terazosin (risk ratio 2.03, 95% CI 1.14 to 3.61, p-value 0.02) and silodosin (risk ratio 2.52, 95% CI 1.15 to 5.52, p-value 0.02).

These studies were generally underpowered but were well-blinded, and there was no obvious risk of bias affecting the results.

Overall, alpha-blockers have a well-established role in treating CP/CPPS, particularly when complaints surround pain and urinary issues. Tamsulosin offers the greatest difference in NIH-CPSI scores with an acceptable side-effect profile. Terazosin, while an effective treatment, needs to be selectively used based on patient profiles and tolerance.

5-Alpha Reductase Inhibitors

5-alpha reductase inhibitors have long been used in the treatment of BPH. The competitive blockade of type II 5-alpha reductase receptors via agents such as finasteride could hypothetically cause the regression of the prostatic epithelium, which is where inflammation is thought to begin, therefore improving voiding and theoretically reducing intraprostatic ductal reflux and intraprostatic pressure. The suppression of angiogenesis decreases blood flow to the gland and could further decrease inflammation in the gland [65]. Three studies in this area were identified and are discussed below [66-69].

Nickel et al. performed an RCT consisting of 64 participants (31 in the placebo arm, 33 in the finasteride 5 mg arm). The study group demonstrated a mean difference in NIH-CPSI scores of -4.60, 95% CI -5.43 to -3.77, but a p-value of >0.05. However, the study was generally underpowered, as highlighted by the results beyond NIH-CPSI scores, and the authors themselves commented that finasteride cannot be used as a monotherapy in CP/CPPS. However, in conjunction with other agents, it may confer benefits. No data were specifically given on pain, QoL, or adverse events [68].

Leskinen et al. performed an RCT involving 41 patients (10 in the placebo group, 31 in the finasteride 5 mg group). The study highlighted statistically significant improvements in pain, International Prostate Symptom Score (IPSS) scores, and NIH-CPSI scores (results noted below) [67]. However, the study was underpowered, as reflected in the lack of statistical significance of the results. It was, therefore, of low quality but did highlight the potential benefits for urinary flow as displayed below in Table 10 [67].

**Table 10:** Results Adapted from Leskinen et al. [67] Qmax=peak urine flow rate in millilitres/second; IPSS=international prostate symptom score *Number of tablets needed of Ketoprofen in the week up to the clinic visit

	Qmax (ml/s)	Residual Volume (ml)	Concomitant Analgesia Usage *	IPPS Pain Score
Finasteride Group				
0 Months	17.7 (1.4)	43 (9)	2.9	4.5
12 Months	21.3 (1.9)	36 (9)	1.9	1.5
P-Value	Not mentioned	Not mentioned	Not statistically significant	<0.001
Placebo Group				
0 Months	20.4 (2.9)	56 (21)	1.2	2.9
12 Months	19.8 (3.4)	59 (29)	1.0	1.5
P-Value	Not statistically significant	Not statistically significant	Not statistically significant	Not statistically significant
P-Value	Not statistically significant	Not statistically significant	Not statistically significant	Not statistically significant

Another study performed in 2004 by Kaplan et al. randomized 64 men with CP/CPPS to either finasteride 5 mg or saw palmetto (a fruit extract) for 12 months. After 12 months, the finasteride group had a mean reduction in NIH-CPSI scores from 23.9 to 18.1 (p<0.003) as compared to the saw palmetto group, which saw scores drop from 24.7 to 24.6 (p=0.41). Peak flows rose from 13.3 to 13.8 in the finasteride arm (p=0.41), but NIH-CPSI scores in urination did not improve to a statistically significant degree. QoL scores, however, improved to a statically significant degree, from 6.9 to 5 (p<0.03), in the finasteride group [69].

Finasteride on the whole was well-tolerated, with only two episodes of reduced libido noted by Kaplan et al. and Nickel et al. and three episodes reported by Leskinen et al. [68-69]. The 5-alpha reductase inhibitors showed some evidence of effectiveness, with mixed results in the domains of urination and QoL but consistent improvements in overall prostatitis scores. As such, a large-scale study needs to be conducted to fully illustrate the effectiveness of this therapy.

Antibiotics

Antibiotics are commonly used in all forms of prostatitis, including cases of abacterial CP/CPPS. It is common for patients who suffer from CP/CPPS to have undergone multiple courses of antibiotics without a causative organism found. In cases where bacteria are isolated in prostatic fluid or other samples, fluoroquinolones and macrolides have been shown to be effective in treating pain [53]. However, as previously suggested, in many cases, no bacteria are found, and it is believed that the therapeutic effect of antibiotics extends beyond anti-microbial effects. In-vitro studies have illustrated reduced expression of IL-6 and IL-8, with ciprofloxacin and levofloxacin decreasing the proliferative activity of mononuclear blood cells, thereby reducing inflammation [53].

Five studies were identified, all relating to patients with NIH-classification type III prostatitis, and the results are outlined in Table 11. These studies suggested a reduction in NIH-CPSI scores (mean difference -2.43, 95% CI -4.72 to -0.15) [51,58,70]. There were no reported adverse events, and generally, the antibiotics were well-tolerated. All of the studies included some variation of combined therapy, which seemed to confer better results, especially with alpha-blockers.

**Table 11:** Selected results of antibiotic therapy vs placebo with respect to change in NIH-CPSI scores CI=confidence interval, NIH-CPSI=National Institutes of Health-Chronic Prostatitis Symptom Index

Study	Antibiotic used in Study	Number of Subjects	Mean Difference vs Placebo (95% CI)	P-Value
Alexander et al 2004 [58]	Ciprofloxacin	87	-2.8 (-5.45 to -0.15)	0.20
Kulovac et al 2007 [53]	Ciprofloxacin	60	-2.4(-4.89 to 0.09)	0.08
Kim et al 2011 [60]	Ciprofloxacin	68	-0.5 (-2.51 to 1.51)	0.05
Nickel et al 2003 [70]	Levofloxacin	80	0.6 (-3.79 to 4.99)	0.20
Wang et al 2016 [51]	Levofloxacin	77	-5.98 (-8.12 to -3.84)	<0.01

The studies that did not add any co-intervention versus a placebo highlighted a mean difference of -1.57 with a 95% CI of -4.77 to 1.63 and a p-value of 0.34 among 167 participants [51,53,58]. Three other papers implemented antibiotics with alpha-blockers as a co-intervention and achieved a mean difference of -2.94 with a 95% CI of -6.2 to 0.37 and a p-value of 0.08 among 205 participants.

The effect of antibiotics on pain was reviewed by Franco et al. and an adapted analysis is seen in Table 12, who suggested that there appeared to be evidence of improvement in this domain [47].

**Table 12:** Antibiotics vs placebo on pain outcomes Adapted from Franco et al. [47] CI=confidence interval; SD=standard deviation; NIH-CPSI=National Institutes of Health-Chronic Prostatitis Symptom Index

Study	Type of Intervention	Intervention: Mean Difference NIH-CPSI Score (SD)	Number of Subjects	Control: Mean Difference NIH-CPSI Score (SD)	Number of Subjects	Mean Difference between Groups (95% CI)	The total weighted mean difference between groups of intervention (95% CI)
Alexander et al 2004 [58]	Ciprofloxacin	-3 (4.6)	42	-1.6 (2.9)	45	-1.4 (-3.03, 0.23)	
Kim et al 2011 [71]	Ciprofloxacin	3.7 (2.8)	28	4 (3.2)	40	-0.3 (-1.73, 1.13)	
							-0.78 (-1.86, 0.3)
Nickel et al 2003 [70]	Levofloxacin	8.5 (5.4)	45	7.9 (4.9)	35	0.6 (-1.66, 2.86)	
Ye et al 2008 [72}	Levofloxacin	4.5 (2.2)	42	6 (2.4)	42	-1.53 (-2.5, 0.55)	
							-0.72 (-2.74, 1.3)
Total							-0.92 (-1.78, -0.06)

These studies were underpowered, and incomplete information was provided regarding random sequencing and masking. Beyond that, reporting bias and performance bias could have affected the results, but the studies suggested that antibiotics as part of a multimodular treatment plan may confer benefits. This suggestion, however, must be balanced against the risk of rising resistance and other potential risks (e.g., C. diff) [58,70-72].

Anti-Inflammatories

The immunological pathophysiology for chronic inflammation in CP/CPPS has been well-researched. Autoimmune mechanisms have been suggested with the recruitment of leukocytes, which trigger the development of CPPS in a way akin to rheumatoid arthritis and IBD [73].

Multiple anti-inflammatory agents have been trialled in the treatment of CP/CPPS. Our search found seven studies that examined corticosteroids, NSAIDs, thiocolchicoside (a muscle relaxant), and ibuprofen.

Overall, the mean difference achieved was -2.50 with a 95% CI of -3.74 to -1.26 among 585 participants from the seven studies [46,54-55,60,74-76]. However, when looking at individual studies, the biggest mean difference achieved was with corticosteroids in Yang et al.’s study involving 158 participants (mean difference -2.82, 95% CI -4.1 to -1.54, p<0.0001). During two courses in combination with levofloxacin, the research team stated that no serious adverse events had occurred and that there was no significant difference in the tolerance between the intervention and the placebo. However, as this study had a complex intervention involving antibiotics, a degree of caution is warranted when interpreting the effects of corticosteroids [55].

In their 21-patient study, Bates et al. did not show any significant changes in NIH-CPSI scores or secondary outcomes; however, their study was underpowered and certainly did not preclude the use of steroids. The study reported that although there were high numbers of eligible patients, many were reluctant to take steroids, given their potential risks [76].

Five studies dealing with NSAIDs were analyzed versus placebo by Franco et al., displaying an overall mean difference of -2.56 with a 95% CI of -4.5 to -0.62 and a p-value of 0.001 involving 369 patients [60,74-76]. The NSAIDs were well-tolerated, with no reported adverse outcomes. Marked improvements in pain (mean difference -2.28, 95% CI -4.08 to -0.48, p-value 0.01, 497 participants) and QoL (mean difference -1.25, 95% CI -2.58 to 0.08, p<0.0001) were suggested [46].

The largest study, by Jiang et al., involved 115 patients taking indomethacin, dexketoprofen, trometamol, or terazosin and achieved a mean difference of -5.66 (-6.81 to -4.5) with a significant p-value of <0.05, but the biggest difference was seen in those taking a combination of dexketoprofen, trometamol, and terazosin [74]. Wu et al. performed a study with celecoxib as an isolated intervention and found statistically significant improvements in NIH-CPSI scores, as well as in the pain, QoL, and urinary sub-scores, which lends credence to the use of NSAIDs [55].

Overall, NSAIDs have a place in the treatment of CP/CPPS, particularly improving pain domains and QoL, with indications of a mild improvement in urinary symptoms. The predominant method is to combine with other agents as a co-intervention and again suggests the importance of tailoring treatments to patients’ needs.

Botulinum Toxin A

Botulinum toxin A has been assessed in two studies listed in Table 13 [77-78]. One by Falahatkar et al. involved 60 participants undergoing intraprostatic Botox injection versus saline and followed the participants for up to six months. Marked reductions were observed in pain scores (mean difference -14.63, 95% CI -16.76 to -12.15, p<0.0001) along with a mean difference in NIH-CPSI scores of -25.80 (95% CI -30.15 to -21.45, p<0.001) and statistically significant improvements in QoL (mean difference -6, 95% CI -7.45 to -4.55, p<0.0001) and urinary domains (mean difference -5.17, 95% CI -6.72 to -3.62, p<0.0001) [77].

**Table 13:** Results of BOTOX as a therapy for CP/CPPS Adapted from Franco et al. [46] CP/CPPS=chronic prostatitis/chronic pelvic pain syndrome; SD=standard deviation; CI=confidence interval; NIH-CPSI=National Institutes of Health-Chronic Prostatitis Symptom Index

Study	Type of Intervention	Intervention- NIH-CPSI Score (SD)	Number of Subjects	Control NIH-CPSI Score (SD)	Number of Subjects	Mean difference Between Groups (95% CI)
Falakhtar et al 2015 [77]	Intraprostatic BOTOX	3.4 (5.6)	30	18 (2)	30	-1.4 (-3.03, 0.23)
Gottsch et al 2011 [78]	Pelvic Floor BOTOX	4.5 (2.2)	42	6 (2.4)	42	-1.53 (-2.5, 0.55)

Pelvic floor Botox injections, however, did not yield the same results, as explained below [78]. The treatments were well-tolerated, with only two cases of mild transient hematuria noted by Falahatkar et al. [77].

Intraprostatic Botox appears to be very promising, and given its adequate tolerance as well as impressive results in the pilot study analyzed, it certainly warrants more investigation. Therefore, at this time, the study group would recommend this treatment as well as larger studies on its effects.

Phytotherapy

Phytotherapy was studied by five teams as highlighted in Table 14. They may reduce prostatitis symptoms compared to a placebo (NIH-CPSI scores: mean difference −5.02, 95% CI -6.81 to -3.23, p<0.001; five studies, 320 participants) and may not be associated with an increased incidence of adverse events (low quality of evidence for which reporting was scant) [47,77-85]. Phytotherapy may not improve sexual dysfunction. There was no information on QoL or anxiety and depression. Further studies on these therapies, particularly Calendula-Curcuma and quercetin, appear to be warranted.

**Table 14:** Franco et al. assessment of various phytotherapy on prostatitis symptoms Adapted from Franco et al. [47] CI=confidence interval

Study	Type of Intervention	Number of Subjects	Mean Difference between Groups (95% CI)	Total Weighted Mean Difference between Groups of Intervention vs Control (95% CI)
Breusov et al 2014 [79]	Prolit Super Septo	57	-9(-16.61, -1.19)	
Morgia et al 2017 [80]	Calendula-Curcuma	48	-6 (-7.28, -4.27)	
Park et al 2005 [81]	Add-on cranberry juice	50	-5.4 (-2.5, 0.55)	
Shoskes et al 1999 [82]	Quercetin	28	-5.8 (-10.8, -0.8)	
Wagnelehner et al 2009 [83]	Pollen extract	137	-2.5(-4.44, -0.56)	
Total				-5.02 (-6.81, -3.23)

When studied by Shoskes et al., quercetin showed statistically significant decreases in NIH-CPSI scores, from 21.0 to 13.1 (p=0.003), versus placebo (20.2 to 18.8, p=0.003) after one month of treatment, with the biggest improvements seen in pain (10.3 to 6.2, p=0.005) and QoL (8 to 4.9, p=0.004) [82]. No such change was observed in the urinary domain.

Calendula-Curcuma suppositories (a mix of turmeric and Asteraceae) were studied by Morgia et al. [80]. Turmeric has long been investigated for its potential analgesic properties alongside its ability to modulate pro-inflammatory cytokines, COX-2, and apoptotic cytokines [85]. In their 48-patient series, Morgia et al. displayed a mean difference in NIH-CPSI scores of -5.5 (p<0.01, SD(standard deviation) 2.31), an improvement in peak flow from 14 ml/s to 17.3 ml/s (p<0.01, SD 4.3), and reduced post-voiding residuals. However, the study was small and relatively short term, with performance bias potentially influencing the results [80].

Cranberry juice, given its low cost and use in the prophylaxis of UTIs, has generated interest for its potential application to CP/CPPS. However, the results were not statistically significant and the study was underpowered. That said, when compared to a placebo, the treatment was well tolerated [81].

Table 15 summarizes the highlights selected results in studies involving phytotherapy:

**Table 15:** Mean difference in pain sub-scores associated with various forms of phytotherapy Adapted from Franco et al. [47] CI=confidence interval

Study	Type of Intervention	Number of Subjects	Mean Difference between Groups (95% CI)	Total Weighted Mean Difference between Groups of Intervention vs Control (95% CI)
Breusov et al 2014 [79]	Prolit Super Septo	57	-4 (-1331, 5.31)	
Morgia et al 2017 [80]	Calendula-Curcuma	48	-2.8 (-5.41, -0.19)	
Park et al 2005 [81]	Add-on Cranberry Juice	50	-1.4 (-2.34 -0.46)	
Shoskes et al 1999 [82]	Quercetin	28	-2.8 (-5.41, -0.19)	
Wagnelehner 2009 [83]	Pollen Extract	137	-1.58 (-2.74, -0.42)	
Total				-1.42 (-1.99, -0.85)

Given its low side-effect profile, ease of availability over the counter, and potential benefits as highlighted by the above results, phytotherapy can form part of a multimodal regime in the treatment of CP/CPPS.

Anti-Depressants

The mental impact of CP/CPPS on patients has been well-documented and has been suggested to form part of the nascence of the disease process itself. Indeed, it constitutes part of the NIH-CPSI questionnaire, and the use of anti-depressants in treating CP/CPPS has been assessed by four studies. These studies, however, suffered from performance bias, were underpowered, and had relatively short follow-up periods.

However, there was a suggestion that there was a place for anti-depressants in the treatment of CP/CPPS, as the results below demonstrate. Sertraline was better tolerated than duloxetine, as shown by Zhang et al. While the results in Table 16 may seem promising when compared to a placebo, none showed statistical significance [86-89].

**Table 16:** Summary of results of anti-depressants in CP/CPPS CP/CPPS=chronic prostatitis/chronic pelvic pain syndrome; SD=standard deviation

Study	Anti-Depressant	Number of Subjects	Length of follow-up	Difference in NIH-CPSI Scores from Baseline (SD)	P-Value
Giannantoni et al 2014 [86]	Duloxetine	38	16 Weeks	-10.03 (+/- 2.2)	<0.01
Zhang et al 2017 [87]	Duloxetine; Sertraline	150	6 Months; 6 Months	-9.54; -14.37	>0.01; <0.01
Lee et al 2005 [88]	Sertraline	14	26 Weeks	-11.7	<0.01
Turkington et al 2002 [89]	Fluvoxamine	42	8 Weeks	Not detailed	<0.01

Phosphodiesterase Inhibitors

Phosphodiesterase inhibitors enhance cavernosal smooth muscle relaxation by blocking the breakdown of cGMP. This aids erectile function and has also been shown to improve lower urinary tract symptoms (LUTs) as there is improved blood flow throughout the pelvic organs, as suggested by Gacci et al. in the context of CP/CPPS.

Three studies were identified that highlighted the use of phosphodiesterase inhibitors in the treatment of CP/CPPS (Table 17) [90-92].

**Table 17:** Summary of results of studies identified as regards the use of phosphodiesterase Inhibitors in the context of NIH-CPSI scores NIH-CPSI=National Institutes of Health-Chronic Prostatitis Symptom Index; SD=standard deviation

Study	Intervention	Number of Subjects	Length of Follow-Up	Difference in NIH-CPSI Scores from Baseline (SD)	P-Value
Benelli et al 2018 [90]	Tadalafil	14	16 weeks	-2.93 (2.43)	<0.000002
Kong et al 2014 [91]	Mirodenafil + Levofloxacin	92	6 weeks	-7.2 (0.1)	<0.05
Cantoro et al 2013 [92]	Sildenafil + Tamsulosin	44	60 days	-9.74 (1.98)	0,574

All three studies looked at phosphodiesterase inhibitors in conjunction with other treatments, which somewhat blurred their true effect. Benelli et al. gave tadalafil 5 mg daily and highlighted a reduction in NIH-CPSI scores, with the biggest reduction in the pain domain. The other two studies combined therapy with other agents. A combination of mirodenafil and levofloxacin resulted in a reduction of 7.2 +/- 0.1 versus a reduction of 3.2 +/- 0.2 with levofloxacin alone. Again, the greatest reduction was seen in the pain domain. In both cases, pain reduction was comparable to NSAIDs [90-92].

However, Cantoro et al. found no statistical difference between sildenafil when combined with tamsulosin and tamsulosin alone. In fact, the greater difference was seen in the monotherapy group (10.54 +/- 1.35 vs 9.74 +/-1.98). There was no mention of domain scores, but their recommendation was that monotherapy was sufficient [90].

These studies were all small, and their follow-ups were no longer than six weeks. Beyond that, only one of these studies directly compared the effect of phosphodiesterase inhibitors with a placebo, and none were double-blinded [90-92].

It is the opinion of this study group that phosphodiesterase inhibitors have a place in the treatment of CP/CPPS, particularly concerning sexual dysfunction and may even have a role in the control of pain and the improvement of urinary symptoms. Larger, longer-term studies, however, are still required.

Cognitive Behavioral Therapy and Anxiolytics

There has been no formal study with published results regarding cognitive behavioural therapy and anxiolytics as of yet. A German group published a feasibility study outlining combined cognitive-behavioural and physiotherapeutic therapy in patients suffering from CPPS [93]. This area requires more research but given its overall holistic benefits and low side-effect profile, it constitutes a reasonable treatment in which pain relief or other mental health indications directs management in this direction.

Anxiolytics is another area for which there are general indications for their implementation in CPPS treatment, but large trials are needed to elucidate their specific effects.

Discussion

CP/CPPS is a syndrome presenting a wide variety of symptoms in patients [7]. As a result, monotherapy often fails. A tailored approach is often more effective. This is supported by numerous trials [49,53,59,80,90-96]. The most widely used questionnaire in the assessment of CP/CPPS is the NIH-CPSI, which has been validated since 1999. While the questionnaire is important in the evaluation of CP/CPPS, clinical phenotyping has been an area of increasing focus.

In this review, we aimed to assess the available evidence and identify effective or promising treatments for CP/CPPS. The review was narrative in nature but aimed to assess the highest level of evidence available in the treatment modalities available. This included treatments beyond those recommended by mainstream guidelines and aimed to broaden the scope of clinician arsenal in caring for this condition. Obviously, as previously mentioned, the narrative nature of this review precludes full data synthesis, but where available, we included data synthesis from relevant meta-analyses. The below table highlights the general quality of evidence assessed in the review. As can be seen, the evidence is generally of moderate to low quality, highlighting the need for further research, particularly in the areas of Botox and physiotherapy. The quality of evidence was judged using the GRADE framework (Grading of Recommendations Assessment, Development and Evaluation) as outlined by Mustafa et al. and outlined in Table 18 [96].

**Table 18:** Level of evidence and assessed quality of studies by intervention

Intervention	Number of studies	Mean Level of Evidence	Quality of Evidence
Acupuncture	4 [15-18]	1b	Moderate
Lifestyle Modification	1 [21]	1b	Low
Physical Activity	1 [22]	1b	Low
Prostatic Massage	4 [22], [25-27]	2b	Low
Extracorporeal shockwave lithotripsy	5 [29-33]	1b	High
Transrectal thermotherapy	3 [35-37]	1b	Low
Physiotherapy	1 [40]	4	Very Low
Transurethral resection of the prostate	1 [44]	4	Very Low
Prostatectomy	3 [41-43]	4	Very Low
Alpha-Blocker	18 [47-65]	1b	Moderate to High
5-Alpha reductase inhibitors	3 [67-69]	1b	Moderate
Antibiotics	5 [51], [53], [58], [60], [70]	1b	Moderate to High
Anti-Inflammatories	7 [46], [54-55], [60], [74-76]	1b	Moderate
Botulinum Toxin A	2 [77-78]	1b	Low to Moderate
Phytotherapy	5 [79-83]	1b	Moderate
Anti-Depressants	4 [86-89]	1b	Moderate
Phosphodiesterase Inhibitors	3 [90-92]	1b	Low

The UPOINT clinical phenotyping approach developed by Shoskes et al. has heralded a new era of treatment approaches for patients [96]. This approach allows practitioners to classify patients according to their symptomology: urinary, psychosocial, organ-specific, infective, neurological, and tenderness in the pelvic floor. With such information, practitioners can focus on treatments for individuals and aim for better control of their condition. Shoskes et al. found that only 22% of patients reported symptoms in a single domain [97]. Additionally, Magri et al. found that a resolution in one domain can lead to a resolution in other domains (even in unrelated domains) [98]. In fact, Magistro et al. reviewed 26 RCTs and concluded that monotherapy is never sufficient to achieve control of CP/CPPS and thus a multifaceted approach is better [7].

Looking at the evidence we have reviewed, our study group feels that a multimodal approach would best serve our patients. The UPOINT framework as proposed by Nickel et al. in 2010 represents an innovation in the tailoring of treatment [94]. Below (Table 19), we present an algorithm to help direct treatments based on our review.

**Table 19:** UPOINT treatment algorithm after review of evidence LUTS=lower urinary tract symptoms; 5-ARI= 5-alpha-reductase inhibitors; ESWL=extracorporeal shockwave lithotripsy; NSAID=non-steroidal anti-inflammatory; IBS=irritable bowel syndrome; Botox=botulinum toxin A

U	P	O	I	N	T	S
Urinary Symptoms	Psychosocial	Organ-Specific	Infection	Neurologic/Systemic	Tenderness	Sexual Dysfunction
Voiding/Storage symptoms	Depression	Prostatic Pain/LUTS	Positive Cultures	Fibromyalgia, IBS, Chronic Fatigue Syndrome	Pelvic floor pain	Erectile Dysfunction, Orgasmic Pain, Retrograde ejaculation/Pain
Alpha-blockers, 5-ARI’s, BOTOX, Acupuncture, Anti-Cholinergics (if overactive bladder)	Anti-Depressants, Anxiolytics (as appropriate)	NSAID’s, Alpha-blockers, 5-ARI’s, ESWL, BOTOX, Phosphodiesterase Inhibitors, Phytotherapy	Antibiotics	Antidepressants, Acupuncture, Phytotherapy	NSAID’s Muscle Relaxants, BOTOX, Physiotherapy, Pelvic Floor Exercises, Acupuncture, ESWL,	Phosphodiesterase Inhibitors, Topical agents

Looking at the non-pharmacological treatments assessed, there was low-quality evidence for many interventions, but most were shown to be safe and to promote general good health. There is evidence to suggest that physical activity, physiotherapy, and lifestyle modification can culminate in improvements in patients with CP/CPPS but can also yield global, mental benefits that can improve CP/CPPS care. TRT may provide pain relief and improve urinary symptoms and QoL, but based on the evidence, it cannot be recommended. ESWL seems to be a promising treatment, reducing NIH-CPSI scores and improving all sub-scores of pain, QoL, and urinary symptoms along with potential improvements in sexual dysfunction. Acupuncture also offers benefits, predominantly in pain relief, and, as such, may form part of the treatment algorithm. Prostatic massage has been anecdotally used as part of treatment for decades; however, based on the evidence reviewed, it cannot be recommended at this time.

Pharmacologically, numerous agents were assessed. Alpha-blockers have a role based on potential benefits concerning urinary symptoms while 5-alpha reductase inhibitors likely reduce symptoms of prostatitis with a tolerable side-effect profile and indications of improvements in urinary symptoms. Anti-inflammatories seem to have a positive effect on pain and certainly have a place in following UPOINT treatment algorithms, but more studies are needed to identify preferred agents. In the systematic review performed by Franco et al., anti-inflammatories showed the greatest improvements, and short-term steroid therapy can be considered coupled with longer-term NSAID therapy [47]. Antibiotics cannot be recommended by the study group despite the potential benefits they confer unless evidence of infection is found. The indiscriminate use of antibiotics is associated with a multitude of problems that, as medical professionals, we should avoid. However, in controlling infections, antibiotics should be used in accordance with bacterial sensitivities.

BOTOX, on the other hand, seems to be very promising, with rather large reductions in NIH-CPSI scores observed. Therefore, a large-scale study should be performed to validate the results, but given its substantial potential benefits and the fact that it is well tolerated by patients, we recommend its judicious implementation, especially in cases where pain cannot be alleviated with standard therapies. It should be noted that global improvements were observed with this treatment. Phytotherapy seems to be beneficial, and given its potential holistic improvements in nutrition status, it is recommended by the study group. Surprisingly, the historic commentary and indeed the obvious nihilism of patients concerning clinical anti-depressants do not convincingly help patients’ scores, lending credence to the claim that focused treatments are more beneficial. Larger studies, however, are required and should form part of the UPOINT arsenal.

The surgical management of chronic prostatitis has fallen out of favour in modern practice. This is reflected in the level and quality of evidence as well as its age. However, a consideration of surgery may be appropriate in very select cases with sufficient counselling, but it should not form part of a treatment algorithm given its permanence and potential morbidity.

## Conclusions

CP/CPPS is a heterogenous term referring to diverse symptomology that requires tailored treatments depending on patients’ complaints. In view of this, we sought to assess the evidence behind treatment options and present a treatment algorithm that was based on the much-discussed UPOINT framework. Future studies should focus on a multimodal therapy based on such a framework and provide future research directions concerning this complex condition.
